# Adipocyte-Specific Protein Tyrosine Phosphatase 1B Deletion Increases Lipogenesis, Adipocyte Cell Size and Is a Minor Regulator of Glucose Homeostasis

**DOI:** 10.1371/journal.pone.0032700

**Published:** 2012-02-28

**Authors:** Carl Owen, Alicja Czopek, Abdelali Agouni, Louise Grant, Robert Judson, Emma K. Lees, George D. Mcilroy, Olga Göransson, Andy Welch, Kendra K. Bence, Barbara B. Kahn, Benjamin G. Neel, Nimesh Mody, Mirela Delibegović

**Affiliations:** 1 Integrative Physiology, University of Aberdeen, Aberdeen, United Kingdom; 2 School of Medical Sciences, University of Aberdeen, Aberdeen, United Kingdom; 3 Department of Experimental Medical Science, Protein Phosphorylation Unit, Lund University, Lund, Sweden; 4 Department of Animal Biology, School of Veterinary Medicine, University of Pennsylvania, Philadelphia, United States of America; 5 Division of Endocrinology, Diabetes and Metabolism, Beth Israel Deaconess Medical Centre, Boston, United States of America; 6 Campbell Family Cancer Research Institute, Ontario Cancer Institute, Princess Margaret Hospital, University of Toronto, Toronto, Canada; Neocodex, Spain

## Abstract

Protein tyrosine phosphatase 1B (PTP1B), a key negative regulator of leptin and insulin signaling, is positively correlated with adiposity and contributes to insulin resistance. Global PTP1B deletion improves diet-induced obesity and glucose homeostasis via enhanced leptin signaling in the brain and increased insulin signaling in liver and muscle. However, the role of PTP1B in adipocytes is unclear, with studies demonstrating beneficial, detrimental or no effect(s) of adipose-PTP1B-deficiency on body mass and insulin resistance. To definitively establish the role of adipocyte-PTP1B in body mass regulation and glucose homeostasis, adipocyte-specific-PTP1B knockout mice (adip-crePTP1B^−/−^) were generated using the adiponectin-promoter to drive Cre-recombinase expression. Chow-fed adip-crePTP1B^−/−^ mice display enlarged adipocytes, despite having similar body weight/adiposity and glucose homeostasis compared to controls. High-fat diet (HFD)-fed adip-crePTP1B^−/−^ mice display no differences in body weight/adiposity but exhibit larger adipocytes, increased circulating glucose and leptin levels, reduced leptin sensitivity and increased basal lipogenesis compared to controls. This is associated with decreased insulin receptor (IR) and Akt/PKB phosphorylation, increased lipogenic gene expression and increased hypoxia-induced factor-1-alpha (*Hif-1α*) expression. Adipocyte-specific PTP1B deletion does not beneficially manipulate signaling pathways regulating glucose homeostasis, lipid metabolism or adipokine secretion in adipocytes. Moreover, PTP1B does not appear to be the major negative regulator of the IR in adipocytes.

## Introduction

Caloric excess and a sedentary lifestyle are major contributors to epidemic obesity levels in Western society. Obesity is associated with complex disorders, including cardiovascular disease and type 2 diabetes [1]. This rising burden of metabolic disease requires the development of new therapeutic strategies.

White adipose tissue (WAT) is the main site for storage of excess energy from food intake, and plays a key role in sensing and coordinating adaptations in whole body glucose metabolism [2], [3]. White adipose tissues function as endocrine and paracrine organs by secreting various adipokines. These bioactive molecules, including leptin, adiponectin, visfatin, omentin, tumor necrosis factor-α (TNF-α), resistin, retinol-binding protein 4 (RBP4) and many others influence metabolic processes such as food intake, glucose- and lipid-metabolism, inflammation and insulin resistance [4]. Insulin resistance precedes the development of type 2 diabetes, and is characterized by reduced insulin-dependent glucose uptake into muscle, adipose tissue and other insulin-sensitive peripheral tissues, inadequate suppression of hepatic glucose production, and accumulation of hepatic lipids [5].

The mechanism(s) leading to insulin resistance remain unclear [6], [7]; however, it is generally agreed that impaired post-insulin receptor (IR) signal transduction is involved [8]. Insulin is secreted from pancreatic β-cells in response to nutrients and transported to target tissues via the circulatory system. Insulin signaling is activated when insulin binds to the IR, located on the plasma membrane. The IR is a heterodimeric complex containing two α-subunits, which enable insulin binding, and two β-subunits, which have inherent tyrosine kinase activity. Once the α-subunits are bound by ligands, the β-subunits can transphosphorylate, which enhances their kinase activity [9]. The activated IR phosphorylates substrates including the insulin receptor substrate (IRS) proteins 1–4, Shc, Cbl and Gab-1 [10]. Upon phosphorylation, the IRS proteins act as docking sites for several src homology region 2 (SH2) domain containing proteins, including the p85 regulatory subunit of phosphatidylinositol 3-kinase (PI3K), which results in PI3K activation [11]. PI3K activation subsequently leads to activation of protein kinase B (Akt/PKB), which has diverse intracellular targets, including glycogen synthase kinase 3 (GSK-3) and the mammalian target of rapamycin (mTOR). Importantly, Akt/PKB is required to promote translocation of GLUT4 to the plasma membrane, and consequently, increase glucose uptake [12].

Protein-tyrosine phosphatase 1B (PTP1B) is a ubiquitously expressed non-receptor tyrosine phosphatase and a key negative regulator of leptin and insulin signaling [13]. PTP1B has a catalytic (PTP) domain, followed by a regulatory region and a membrane localization domain, which tethers the enzyme to the cytoplasmic face of the endoplasmic reticulum (ER) [14], [15]. Global PTP1B^−/−^ mice exhibit reduced adiposity, enhanced tyrosine phosphorylation of the IR in muscle and liver – but not adipose tissue – and display improved glucose homeostasis as a consequence of increased systemic insulin sensitivity [16], [17]. The increased insulin sensitivity in PTP1B^−/−^ mice is tissue-specific, as glucose uptake is elevated in muscle, but not in adipose tissue [16]. PTP1B tissue-specific knockout mice with deletions in brain, liver or skeletal muscle were generated to investigate the site(s) and mechanism(s) of PTP1B action in the regulation of insulin sensitivity and body mass/adiposity [5], [18], [19]. Neuronal-specific PTP1B knockout mice, despite increased leptin levels, exhibit reduced body mass and adiposity due to leptin hypersensitivity, resulting in reduced food intake and increased energy expenditure [18]. Furthermore, mice lacking PTP1B specifically in pro-opiomelanocortin (POMC) neurons display reduced adiposity, improved leptin sensitivity, increased energy expenditure and improved glucose homeostasis on a high-fat diet (HFD) compared with wild-type mice [20]. Muscle- or liver-specific PTP1B knockouts have comparable body weight and adiposity to controls. However, they exhibit improved insulin sensitivity and glucose tolerance [5], [19]. Mice with an adipose-deficiency of PTP1B were also generated by using the adipocyte protein 2 (aP2)-promoter cassette to drive Cre recombinase expression. These aP2-crePTP1B^−/−^ mice showed increased body weight on a HFD compared with littermate controls [18]. However, whether adipocyte-PTP1B was the sole cause of the observed weight gain in these mice is unclear, as the aP2 promoter is known to delete in cell types other than adipocytes [21], [22].

Although the negative regulatory effects of PTP1B activity on insulin action have been well documented in the muscle and liver, studies on the role of PTP1B in adipocytes have provided ambiguous results. Insulin-stimulated IR phosphorylation is unchanged in the adipose tissue of PTP1B^−/−^ mice [17]. However, over-expression of PTP1B in 3T3-L1 adipocytes inhibits insulin-stimulated phosphorylation of the IR and IRS-1, and decreases PI3K activation [23], [23,24]. Ruffolo *et al.* [25] demonstrated increased basal (*ad libitum* fed/non-insulin stimulated) phosphorylation of p70S6K at site Thr-389 in isolated adipocytes from global PTP1B^−/−^ mice, compared with controls. They suggested that the enhanced basal phosphorylation of p70S6K, which decreased IRS-1 levels, was the cause of reduced glucose uptake into isolated adipocytes from PTP1B^−/−^ mice and that adipose-PTP1B deletion causes tissue-specific insulin resistance. By contrast, antisense oligonucleotides, which lower PTP1B levels only in adipose tissue and liver, reduce diet-induced obesity and improve insulin signaling in obese (*ob/ob*) and diabetic (*db/db*) mice [26], [27]. It is not clear whether these effects reflected loss of PTP1B in adipose tissue or liver (or both). Here we used mice expressing Cre recombinase under the control of an adiponectin-promoter cassette [28] – which is exclusively expressed in adipocytes [29] – to delete PTP1B, and thereby definitively establish the physiological and molecular consequences of adipocyte-specific PTP1B deletion *in vivo*.

## Materials and Methods

### Ethics statement

All animal procedures were approved by the University of Aberdeen Ethics Review Committee Board and performed under a project license approved by the Home Office under the Animals (Scientific Procedures) Act 1986 (PPL60/3951).

### Animal studies

PTP1B^fl/fl^ mice [18] and mice expressing Cre recombinase (Cre) under the control of the adiponectin promoter [28] were described previously; the latter were generously provided by Dr Evan Rosen, Beth Israel Deaconess Medical Centre, Harvard Medical School, Boston, USA. To generate adipocyte-specific PTP1B^−/−^ mice, PTP1B^fl/fl^ mice were crossed with adiponectin-cre mice. DNA extraction and genotyping for the PTP1B floxed allele and the presence of Cre by PCR were performed as described previously [18]. Sequences of primers used for PCR are provided in the appendix (Table S1). Mice studied were age-matched littermates, which had been backcrossed to pure C57BL/6 mice for seven generations. For most studies, mice were housed in groups and maintained at 22–24°C on a 12-h light/dark cycle with free access to food and water. To measure food intake, mice were caged individually and the weight of food consumed was measured over a four-week period. For insulin signaling experiments, 25-week old chow- or HFD-fed mice were fasted overnight and then injected intraperitoneally with saline or insulin (10 mU/g body weight). After 10 minutes, mice were sacrificed by cervical dislocation. Tissues were dissected immediately and frozen in liquid nitrogen.

### Body composition

At weaning (21 days), mice were placed on standard 3.4% fat chow pellet diet (Rat and Mouse Breeder and Grower, Special Diets Services, DBM, Scotland) or HFD (Adjusted Calories Diet, 55% fat, Harlan Teklad, USA) and weight was recorded weekly. The approximate fatty acid profile of Adjusted Calories Diet (% total fat) was 28% saturated, 30% trans, 28% monounsaturated (cis) and 14% polyunsaturated (cis), as described previously [30]. Adiposity was measured at the end of the study by individually weighing dissected fat pads.

### Isolation of peritoneal macrophages

Mice were sacrificed by cervical dislocation. DMEM (Gibco, Paisley, UK) was injected intraperitoneally, mice were agitated, and medium containing macrophages was collected. Peritoneal macrophages were maintained for 24–48 hours at 37°C in a humidified atmosphere with 5% CO_2_.

### Isolation and maturation of bone marrow derived macrophages

Mice were sacrificed by cervical dislocation. Bone marrow derived macrophages were obtained by flushing out femurs and tibiae with DMEM (Gibco). Cells were passed through 19 G needles to disrupt the bone marrow plugs and centrifuged at 900 *g* for 5 min. Bone marrow mononuclear phagocyte precursor cells were matured for seven days in culture medium (DMEM (Gibco), 10% FBS (Invitrogen, Paisley, UK), 100 U/ml penicillin (Gibco) and 100 mg/ml streptomycin (Gibco)) supplemented with 20% L929-cell conditioned medium in untreated polystyrene Petri dishes. Bone marrow derived macrophages (BMDM) were maintained at 37°C in a humidified atmosphere with 5% CO_2_.

### Histology

Epididymal, subcutaneous and peri-renal adipose tissue was fixed in formaldehyde, embedded in paraffin, sectioned, and stained with hematoxylin and eosin. Average adipocyte diameter was quantified by measuring 50 adipocytes at 20× magnification, as described previously [31]. Scale bars were determined by measurement with a scale calibrator (Stage Micrometer, Graticules Ltd, Kent, England).

### Metabolic measurements

Tail blood glucose from fasted (5-h or 16-h) mice was measured by using glucometers (Accu-Chek, Burgess Hill, UK). Serum insulin, leptin (CrystalChem, Downers Grove, USA), adiponectin (Millipore) and TNF-α levels (R&D Systems, Minneapolis, USA) were determined by ELISA. Serum RBP4 was determined by immunoblotting. Glucose and insulin concentrations were used to calculate the homeostasis model assessment of insulin resistance (HOMA-IR), a reliable marker of insulin sensitivity [32], which is defined as: fasting glucose (mg/dl)×fasting insulin (µU/ml)/405. Serum glucose and triglycerides were determined using appropriate kits (Sigma, Gillingham, UK). Glucose tolerance tests (GTT) were performed as described previously [16], [33]. Insulin tolerance tests (ITT) were performed on fasted (5-h) mice by measuring blood glucose values immediately before and at 15, 30, 60, and 120 min after intraperitoneal injection of insulin (chow 0.6 mU/g body weight and HFD 1.1 mU/g body weight; Humulin R, Eli Lilly Corp., Indianapolis, USA).

### Leptin sensitivity

Mice were caged individually with free access to food and water. Food intake and body weight were monitored daily throughout the study. Mouse leptin (R&D Systems) was administered to chow- and HFD-fed adip-crePTP1B^−/−^ and control littermates intraperitoneally twice daily for three days (morning and evening dose, 0.5 µg/g; total dose for 24-h period, 1.0 µg/g).

### Positron emission tomography (PET) scanning

PET scanning was carried out using the glucose analog, 2-deoxy-2-(^18^F)fluoro-D-glucose (FDG), a marker of glucose metabolism, which was manufactured in the radiochemistry facility at the University of Aberdeen. FDG administration was performed in conscious, fasted (8-h) mice which had free access to water. Mice were kept at 35°C by placing cages on a heating pad. Warming started at least 30 min before FDG injection and continued during the FDG uptake period. Mice were conscious during the FDG uptake with cages kept in the dark. In the pre-imaging period, FDG (range: 17.8–22.1 MBq) was intraperitoneally injected (injected volume 0.5 ml). The uptake occurred outside the scanner (in the cage) for 45 min, during which time the mice were placed on a running wheel. Running was voluntary and the time that each mouse exercised was recorded. Mice were anesthetized with isofluorane (1.5–2.0% with 2 l/min O_2_), and were placed on the bed of the scanner in the supine position (head first). The body and the head of the mouse were secured to the bed with tape. A CT scan was obtained first, followed by a 40 min PET. Emission data was collected using a SEDECAL Argus dual-ring PET scanner (Madrid, Spain), in a temperature-controlled room. A complete performance evaluation of the SEDECAL Argus dual-ring PET scanner has been performed, as described previously [34]. Corrections for attenuation, random coincidence and photon scatter were applied and the images were reconstructed using Fourier Rebinning and a 2D ordered subsets expectation maximization algorithm, supplied by the manufacturer. The images were converted into semi-quantitative units (SUVs) by dividing the uptake by the injected activity and multiplying by the weight of mice. Analysis was performed by drawing regions of interest on the registered PET and CT images and calculating the ratio of activity in the brain, muscle and brown adipose tissue. For each tissue, the region of interest (ROI) was defined in a single mouse and copied to the scans from the other mice.

### Adipocyte isolation

All chemicals were from Fisher Scientific (Loughborough, UK) (unless stated otherwise). Mice were sacrificed by cervical dislocation. Fresh epididymal adipose tissue was digested at 37°C in a shaking water bath (100 rpm) for 60 minutes in Krebs-Ringer-Hepes (KRH) buffer pH 7.4 which contained 125 mM NaCl, 5 mM KCl, 1 mM KH_2_PO_4_, 2.5 mM MgSO_4_, 2.5 mM CaCl_2_ 2H_2_O, 2 mM glucose, 25 mM HEPES, 3.5% BSA (Cohn Fraction V), 200 nM adenosine (Sigma) and 1 mg/ml type I collagenase (244 U/mg) (Worthington Biochemical, Lakewood, USA). The digest was filtered through cotton mesh to remove debris and washed three times in KRH buffer without collagenase. At the final wash, 2 ml of dinonyl phthalate oil (Sigma) was added to the adipocytes, which were then centrifuged for 5 min at 800 rpm. The top layer of adipocytes were transferred to micro-centrifuge tubes and centrifuged for 1 min at 3000 rpm. The infranatant was removed with 19 G×1 1/2 syringes. Adipocyte lysates were then prepared in radioimmunoprecipitation assay (RIPA) buffer containing fresh sodium orthovanadate and protease inhibitors, as described previously [19].

### Lipogenesis assay

The assay was performed as described previously [35]. Adipocytes were digested in KRH buffer pH 7.4 and filtered, as described above, but washed three times and resuspended in a low glucose variant of KRH buffer containing 0.55 mM glucose. The adipocytes were resuspended at a 2% packed cell volume, which was determined as described previously [36]. The assay was performed in quadruplicate. 700 µl of the 2% adipocyte cell suspension was added to each tube and incubated for 1-h at 37°C with 14 µl of 22 µCi/ml tritiated glucose (D-[6-3H]-glucose, Perkin Elmer, Cambridge, UK) and 0, 1, 3, 10, 30 and 100 nM insulin concentrations. After the incubation, the assay was stopped by adding 3.5 ml of 2,5-diphenyloxazole (Sigma) and 1,4-bis(5-phenloxazol-2-yl)benzene (Sigma) toluene-based scintillation liquid (Sigma). A zero sample was also included in the experiment to measure how much glucose ends up in the lipid phase during extraction without having been used for lipid synthesis. This was done by adding 700 µl of the 2% adipocyte cell suspension to scintillation tubes containing 14 µl of 22 µCi/ml tritiated glucose which was then stopped immediately by adding 3.5 ml PPO-POPOP toluene-based scintillation liquid.

### Immunoblotting

Tissue lysates were prepared in RIPA buffer containing fresh sodium orthovanadate and protease inhibitors, as described previously [19]. Proteins were separated by 3–8%, 10% or 4–12% SDS-PAGE and transferred to nitrocellulose membranes. Immunoblots were performed using antibodies from Cell Signaling (Cell Signaling by NEB, Hitchin, UK) (unless stated otherwise) against pIR Y1158, pIRS-1 S636/639, total IRS-1, pAkt/PKB S473, total Akt/PKB, pERK1/2 MAPK T202/Y204, pS6 ribosomal protein S235/236, pS6 ribosomal protein S240/244, p-p70S6K T389, total p70S6K, p-mTOR S2448, total mTOR (Santa Cruz, Insight Biotechnology, Wembley, UK) pGSK-3α S21, pGSK-3β S9, pAMPK T172, total AMPKα, pIR Y1162/63 (Invitrogen), total IR (Santa Cruz), SHP2 (Santa Cruz), pIRS-1 Y608 (CalBiochem), RBP4 (Dako, Cambridgeshire, UK), TC-PTP (R&D Systems) and PTP1B (Millipore, Chandlers Ford, UK). Immunoblots were developed with horseradish peroxidase-conjugated secondary antibodies, visualized using enhanced chemiluminescence, and quantified by densitometry scanning with Image J or Bio1D software (PeqLab, Fareham, UK).

### Immunoprecipitations

Tissue lysates were prepared in RIPA buffer containing fresh sodium orthovanadate and protease inhibitors as described previously [19]. IR was immunoprecipitated by adding 1 µg of IR antibody (Santa Cruz) to each 200 µg sample of epididymal adipose tissue lysate, and then incubated overnight in an Eppendorf Thermomixer (Eppendorf UK Ltd, Cambridge, UK) at 4°C with constant shaking at 550 rpm. Protein-A Sepharose beads were washed 4 times in buffer containing 50 mM Tris/HCl pH 7.5 and 150 mM NaCl before being resuspended in RIPA at 1∶1 (beads/buffer, v/v). Thirty (30) µl of bead slurries were added to each immunoprecipitation sample, which was then incubated with gentle end-over-end mixing for 2-h at 4°C. Proteins from the immunoprecipitates were resolved by 10% SDS-PAGE, transferred to nitrocellulose membranes, and immunoblots were performed as described above, using mouse anti-phospho tyrosine antibodies (Cell Signaling).

### Ex vivo insulin signaling

Adipocytes were digested in KRH buffer pH 7.4 and filtered, as described above, and then resuspended in a 10% cells/KRH buffer suspension. The 10% adipocyte suspensions were aliquoted into 1.5 ml tubes containing a final concentration of 0 nM, 1 nM, 10 nM or 100 nM insulin. Adipocytes were incubated in a water bath for 10 minutes at 37°C with gentle shaking. Infranatant was removed from adipocyte cell suspensions and the remaining adipocytes were lysed in RIPA buffer containing fresh sodium orthovanadate and protease inhibitors, as described previously [19].

### Gene expression analysis

Total RNA was isolated from mouse epididymal adipose tissue using TRI Reagent (Ambion, Warrington UK), according to the manufacturer's protocol. First strand cDNA was synthesized from 1 µg of total RNA employing the Bioline Bioscript™ Pre-amplification System and oligo(dT)_12–18_. Four (4) µl of diluted cDNA (1∶100) was used to amplify target genes by real-time RT-PCR (20 µl), using GoTaq qPCR Master Mix (Promega, Southampton, UK). The Roche LightCycler® 480 System (Roche Diagnostics, Burgess Hill, UK) was used for analysis. Relative gene expression was calculated using the comparative Ct (2−ΔΔCt) method. The relative copy numbers of mouse hypoxanthine-guanine phosphoribosyltransferase (*Hprt*) mRNA was used for normalization. PCRs were followed by melting curves (60–95°C). Sequences of primers used real-time quantitative PCR are provided in the appendix (Table S1).

### Data analysis

Data are expressed as mean ± SEM. Statistical analyses were performed using one-way ANOVA with Tukey's multiple comparison post tests, two-way ANOVA with Bonferroni multiple comparisons post tests, and two-tailed Student's t tests, as appropriate. *P*≤0.05 was considered statistically significant. GraphPad Prism 5 and SPSS Version 17 statistical software were used for analyses.

## Results

### Adipocyte-specific deletion of PTP1B increases adipocyte cell size but does not affect body mass, adiposity or food intake

**Figure 1:**
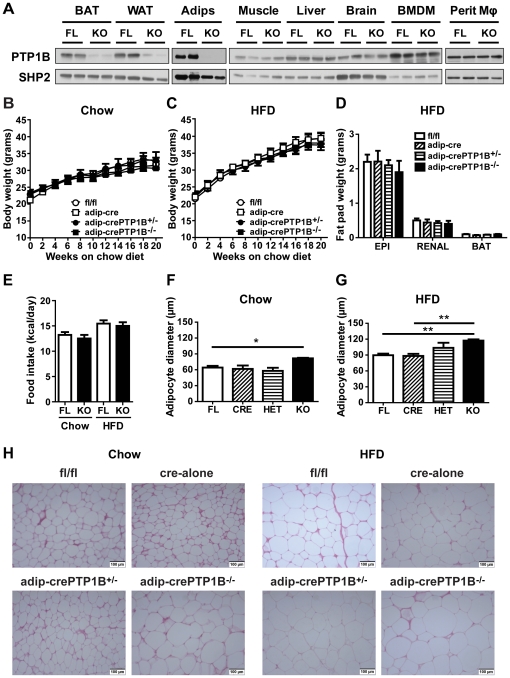
Adipocyte-specific deletion of PTP1B has no effect on body mass, adiposity or food intake. *A*: Deletion efficiency of PTP1B in fl/fl controls (FL) and adip-crePTP1B^−/−^ mice (KO), as detected by immunoblotting. Tissues shown, (left to right) are brown adipose tissue (BAT), white adipose tissue (WAT), isolated adipocytes (Adips), muscle, liver, brain, bone marrow derived macrophages (BMDM) and intraperitoneal macrophages (Perit Mϕ). *B*: Weight curves for fl/fl (*n* = 14), adip-cre (*n* = 6), adip-crePTP1B^+/−^ (*n* = 5) and adip-crePTP1B^−/−^ mice (*n* = 4) on chow diet for 21 weeks from weaning. *C*: Weight curves for fl/fl (*n* = 13), adip-cre (*n* = 6), adip-crePTP1B^+/−^ (*n* = 5) and adip-crePTP1B^−/−^ mice (*n* = 4) on HFD diet for 21 weeks. *D*: Fat pad weight of mice on HFD for 21 weeks. Tissues shown, (left to right) are epididymal (EPI), peri-renal (RENAL) and brown adipose tissue (BAT). Fl/fl (*n* = 13); adip-cre (*n* = 6); adip-crePTP1B^+/−^ (*n* = 5); adip-crePTP1B^−/−^ (*n* = 4). *E*: Daily food intake of mice on chow or HFD. fl/fl (FL) (chow *n* = 5, HFD *n* = 7); adip-crePTP1B^−/−^ (KO) (chow *n* = 4, HFD *n* = 5). *F*: Increased epididymal adipocyte cell size in chow-fed adip-crePTP1B^−/−^ mice (KO) compared with adip-crePTP1B^+/−^ mice (HET), fl/fl (FL) and cre-alone controls (CRE). *G*: Increased epididymal adipocyte cell size in HFD-fed adip-crePTP1B^−/−^ mice (KO) compared with adip-crePTP1B^+/−^ mice (HET), fl/fl and cre-alone controls (FL/CRE). *H*: Hematoxylin and eosin stained epididymal white adipose tissue of mice on chow or HFD diet for 21 weeks. *n* = 4 mice/group. White circles = fl/fl; white squares = adip-cre; black circles = adip-crePTP1B^+/−^; black squares = adip-crePTP1B^−/−^. White bars = fl/fl; diagonally striped bars = adip-cre; horizontally striped bars = adip-crePTP1B^+/−^; black bars = adip-crePTP1B^−/−^. Data are represented as mean ± SEM. Data were analyzed using two-tailed Student's t test (**P<0.01).

Mice with an adipocyte-specific deletion of PTP1B (hereafter termed adip-crePTP1B^−/−^ mice) were generated by crossing PTP1B^fl/fl^ mice (hereafter termed fl/fl mice) to transgenic mice with a knock-in of Cre into the adiponectin locus [28]. To account for potential effects of adiponectin gene dosage or Cre expression, mice heterozygous for adipocyte-specific PTP1B deletion and adiponectin-cre-alone mice (hereafter termed adip-crePTP1B^+/−^ and adip-cre mice, respectively) were included in the study. Adip-crePTP1B^−/−^ mice lack PTP1B in brown adipose tissue (BAT), WAT and isolated white adipocytes but not in other tissues (Figure 1A). WAT from adip-crePTP1B^+/−^ mice also had decreased PTP1B levels (∼50%) as expected from a heterozygous deletion (Figure S1). PTP1B levels were normal in fl/fl and adip-cre mice (Figure 1A and Figure S1). Importantly, there was no deletion of macrophage-PTP1B in adip-crePTP1B^−/−^ mice (Figure 1A), which is a potential concern in studies that use aP2-cre mice to achieve deletion in adipocytes [21]. All groups of mice were placed onto either chow (3.4% fat) or HFD (55% fat) after weaning. Adip-crePTP1B^−/−^ and fl/fl control mice displayed similar body weights (Figure 1B) and lengths (data not shown) on chow diet. All groups of mice gained more weight on HFD than on chow, but body weights (Figures 1B and C) and lengths (data not shown) were comparable between genotypes. Adiposity (Figure 1D) and food intake (Figure 1E) were also similar in all groups of mice on chow and HFD. Interestingly, hematoxylin and eosin staining of epididymal adipose tissue sections revealed significant increases in adipocyte cell size in chow- and HFD-fed adip-crePTP1B^−/−^ mice compared with their respective controls (Figures 1F, G and H). The increased adipocyte cell size appears to be depot specific as there were no cell size differences in subcutaneous or peri-renal fat pads between HFD-fed adip-crePTP1B^−/−^ and fl/fl control mice (Figure S4). The increased size also appears to be dose specific as adip-crePTP1B^+/−^ mice only displayed a slight trend towards enlarged adipocytes on a HFD (Figure 1G and H).

### Mild glucose intolerance/insulin resistance in HFD-fed adip-crePTP1B^−/−^ mice

**Figure 2:**
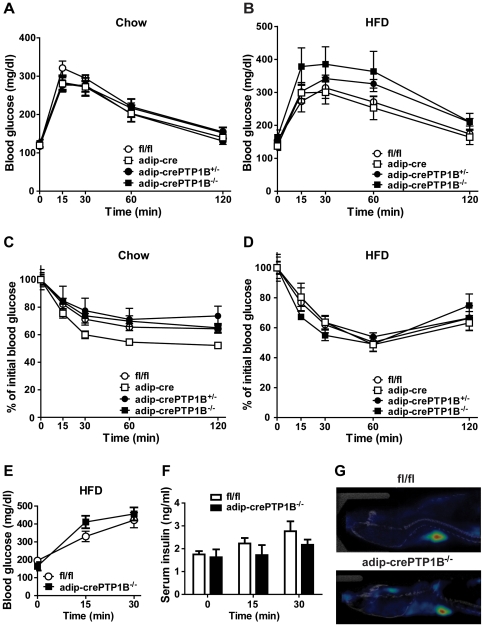
Glucose homeostasis in adipocyte-specific PTP1B knockout mice. *A*: GTT (2 mg/g glucose) of mice on chow diet for 20 weeks from weaning fl/fl (*n* = 6); adip-cre (*n* = 6); adip-crePTP1B^+/−^ (*n* = 5); adip-crePTP1B^−/−^ (*n* = 3). *B*: GTT (1.5 mg/g glucose) of mice on HFD for 19 weeks. fl/fl (*n* = 7); adip-cre (*n* = 6); adip-crePTP1B^+/−^ (*n* = 5); adip-crePTP1B^−/−^ (*n* = 4). *C*: ITT (Insulin 0.6 mU/g body weight) of mice on chow diet for 21 weeks from weaning. fl/fl (*n* = 8); adip-cre (*n* = 6); adip-crePTP1B^+/−^ (*n* = 5); adip-crePTP1B^−/−^ (*n* = 4). *D*: ITT (Insulin 1.1 mU/g body weight) of mice on HFD for 20 weeks fl/fl (*n* = 8); adip-cre (*n* = 6); adip-crePTP1B^+/−^ (*n* = 5); adip-crePTP1B^−/−^ (*n* = 4). *E:* GTT (1.5 mg/g) of mice on HFD. fl/fl (*n* = 5); adip-crePTP1B^−/−^ (*n* = 5). Glucose-stimulated insulin secretion of HFD-fed mice during GTT. fl/fl (*n* = 5); adip-crePTP1B^−/−^ (*n* = 5). *G:* Representative PET scan image from fl/fl (top panel) and adip-crePTP1B^−/−^ (bottom panel) mice. White circles = fl/fl; white squares = adip-cre; black circles = adip-crePTP1B^+/−^; black squares = adip-crePTP1B^−/−^. White bars = fl/fl; black bars = adip-crePTP1B^−/−^. Data are represented as mean ± SEM.

Compared to fl/fl controls, adip-crePTP1B^−/−^ mice displayed significantly higher fasted glucose levels at 8 and 14 weeks HFD, with no differences in fasted serum insulin (Table 1). The homeostasis model assessment of insulin resistance (HOMA-IR) was significantly higher in adip-crePTP1B^−/−^ mice compared with fl/fl controls after 14 weeks of HFD (Table 1). Glucose (GTT) and insulin (ITT) tolerance tests were performed on chow- and HFD-fed adip-crePTP1B^−/−^ mice to assess whole body glucose homeostasis. There were no significant differences in glucose tolerance or insulin sensitivity between adip-crePTP1B^−/−^ mice and fl/fl controls on chow (Figures 2A and C) or HFD (Figures 2B and D). The area under the curve of Figure 2B was also not significantly different (Figure S3); in addition, there were no significant differences in glucose-stimulated insulin secretion between adip-crePTP1B^−/−^ mice and fl/fl controls during the GTT (Figures 2E and F). Serum adiponectin, RBP4 and triglyceride levels were comparable in adip-crePTP1B^−/−^ mice and fl/fl controls (Table 1). Serum TNF-α concentrations were below the level of detection of the mouse ELISA in most control and adip-crePTP1B^−/−^ mice on both chow and HFD (data not shown). To further determine the flux of glucose in various insulin-sensitive tissues including brain, muscle and BAT, PET scans were performed on fl/fl and adip-crePTP1B^−/−^ mice using 2-deoxy-2-(18F)-fluoro-D-glucose (FDG). In agreement with other *in vivo* metabolic tests, there were no significant differences in the uptake and metabolic activity of glucose in brain, muscle or BAT between fl/fl and adip-crePTP1B^−/−^ mice (Figures 2G and Table 2).

**Table 1:** Metabolic parameters in fasted fl/fl, adip-cre, adip-crePTP1B^+/−^ and adip-crePTP1B^−/−^ mice.

Parameter	fl/fl	adip-cre	adip-crePTP1B^+/−^	adip-crePTP1B^−/−^
**Blood Glucose (mg/dl)**				
Chow 14 Weeks	63.5±7.9	51.2±7.7	71.8±7.1	63.6±7.3
HFD 8 Weeks	99.9±6.1	115.8±8.8	114.6±5.6	148.4±21.9 *
HFD 14 Weeks	64.7±8.9	96.5±11.9	90.7±19.3	118.4±13.1 *
**Serum Insulin (ng/ml)**				
Chow 14 Weeks	0.1±0.02	0.17±0.03	0.16±0.03	0.08±0.02
HFD 8 Weeks	1.56±0.22	2.1±0.31	1.56±0.05	1.29±0.37
HFD 14 Weeks	2.42±0.5	2.9±0.51	2.34±0.28	3.23±1.02
**HOMA-IR** a				
Chow 14 Weeks	0.37±0.08	0.44±0.07	0.62±0.14	0.24±0.06
HFD 8 Weeks	8.3±1.1	12.93±2.15	10.11±0.9	11.29±0.53
HFD 14 Weeks	7.87±1.57	15.14±3.61	11.87±3.44	19.51±5.38 *
**Serum Leptin (ng/ml)**				
Chow 14 Weeks	0.93±0.16	1.46±0.58	1.27±0.53	1.95±0.55 †
HFD 8 Weeks	0.68±0.18	1.85±0.58	1.52±0.31	4.60±1.16 *
HFD 14 Weeks	2.61±1.05	6.46±1.59	4.82±0.51	6.81±2.21
**Serum Adiponectin (µg/ml)**				
Chow 21 Weeks	15.67±2.11	11.92±0.99	15.10±1.73	16.84±1.55
HFD 21 Weeks	15.50±1.86	13.99±1.26	16.95±1.46	15.53±1.13
**Serum RBP4 (AU)**				
Chow 21 Weeks	1.0±0.03	ND	ND	1.02±0.05
HFD 21 Weeks	0.94±0.07	ND	ND	0.82±0.07
**Serum Triglycerides (mg/dl)**				
Chow 21 Weeks	82.2±9.1	78.4±12.0	91.6±22.6	95.0±22.4
HFD 21 Weeks	128.6±13.2	106.6±19.4	123.9±20.4	97.9±14.2
**Serum Free Fatty Acids (mM)**				
Chow 21 Weeks	1.34±0.1	1.13±0.14	0.96±0.25	0.8±0.18
HFD 8 Weeks	1.82±0.18	1.37±0.15	1.96±0.21	1.45±0.17

a HOMA-IR, homeostasis model assessment of insulin resistance. Data are means ± SEM and were analyzed by one-way ANOVA with Tukey's multiple comparison post tests or two-tailed student's t test.

*P<0.05;

† = 0.09 for the indicated genotype compared with PTP1B fl/fl control littermates. *n* = 4–14 mice/group.

**Table 2:** PET scan average standardized uptake values from fl/fl and adip-crePTP1B^−/−^ mice.

Genotype	Brain (SUV)	Muscle (SUV)	BAT (SUV)
fl/fl	0.20±0.02	0.39±0.02	0.62±0.34
adip-crePTP1B^−/−^	0.18±0.02	0.35±0.02	0.64±0.27

Data are means ± SEM. *n* = 3–4 mice/group.

### Increased serum leptin levels and reduced leptin sensitivity in HFD-fed adip-crePTP1B^−/−^ mice

**Figure 3:**
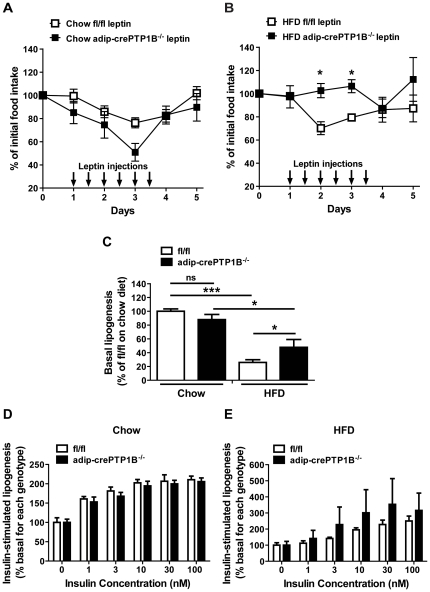
Decreased leptin sensitivity and increased lipogenesis in HFD-fed adip-crePTP1B^−/−^ mice. *A*: Leptin sensitivity, as measured by the percentage change in food intake after leptin administration in chow-fed adip-crePTP1B^−/−^ (*n* = 5) and fl/fl control mice (*n* = 5). *B*: Leptin sensitivity in HFD-fed adip-crePTP1B^−/−^ (*n* = 3) and fl/fl control mice (*n* = 3). *C*: Basal lipogenesis in chow and HFD-fed adip-crePTP1B^−/−^ (*n* = 3) and fl/fl control mice (*n* = 3). *D*: Insulin-stimulated lipogenesis of chow-fed adip-crePTP1B^−/−^ (*n* = 4) and fl/fl control mice (*n* = 4). *E*: Insulin-stimulated lipogenesis of HFD-fed adip-crePTP1B^−/−^ (*n* = 3) and fl/fl control mice (*n* = 3). White squares = chow fl/fl leptin; white triangles = HFD fl/fl leptin; black squares = chow adip-crePTP1B^−/−^ leptin; black triangles = HFD adip-crePTP1B^−/−^ leptin. White bars = fl/fl; black bars = adip-crePTP1B^−/−^. Data are represented as mean ± SEM; Data were analyzed using a two-way ANOVA with Bonferroni multiple comparisons post-tests (*P≤0.05; ***P<0.001).

At eight weeks HFD, leptin levels were three- to seven-fold higher in adip-crePTP1B^−/−^ mice compared with adip-cre and fl/fl controls, respectively (Table 1), suggesting increased leptin secretion with adipocyte-PTP1B deficiency and potential development of leptin resistance. Since brain-specific PTP1B^−/−^ mice also exhibited increased leptin secretion but remained leptin sensitive [18], we directly assessed leptin sensitivity in these mice. Adip-crePTP1B^−/−^ and fl/fl control mice were injected twice a day with saline or leptin for three days and food intake and body weight were monitored. Compared with controls, chow-fed adip-crePTP1B^−/−^ mice displayed no significant differences in response to leptin administration (Figure 3A), but HFD-fed adip-crePTP1B^−/−^ mice were significantly more resistant than controls to leptin administration, which was most apparent on day two and three of leptin injections (Figure 3B). There were no significant effects of saline control injections on food intake in chow fed adip-crePTP1B^−/−^ and fl/fl control mice (Figure S2).

### Adipocyte-specific PTP1B deletion increases the basal lipogenic rate but does not affect insulin-stimulated lipogenesis in HFD-fed adip-crePTP1B^−/−^ mice

Since insulin and leptin are known to regulate lipogenesis [37]–[39], we performed lipogenesis assays in our mice to directly determine whether adipocyte-PTP1B deletion affects adipocyte insulin sensitivity, and to investigate the underlying cause(s) of increased adipocyte cell size. To evaluate the ability of adip-crePTP1B^−/−^ and fl/fl control and mice to basally metabolize glucose into fatty acids, and subsequently triglycerides, (giving an indication of the level/activity of lipogenic enzymes) basal lipogenic rates were determined. On a chow diet, basal lipogenesis was not significantly different between adip-crePTP1B^−/−^ and control mice (Figure 3C). However, on a HFD, adip-crePTP1B^−/−^ mice displayed significantly higher basal lipogenesis compared with fl/fl controls (Figure 3C), consistent with the increase in adipocyte size (Figures 1F, G and H). To evaluate the insulin-sensitivity of adipocytes, a radioactive insulin-stimulated lipogenesis assay was performed on isolated adipocytes from fl/fl and adip-crePTP1B^−/−^ mice, on chow and HFD. On both chow and HFD, isolated adipocytes from adip-crePTP1B^−/−^ mice displayed no differences in the insulin-stimulated lipogenic response compared with adipocytes from fl/fl control mice (Figures 3D and E), suggesting no differences in glucose uptake.

### Decreased in vivo insulin signaling in adipose tissue from HFD-fed adip-crePTP1B^−/−^ mice

**Figure 4:**
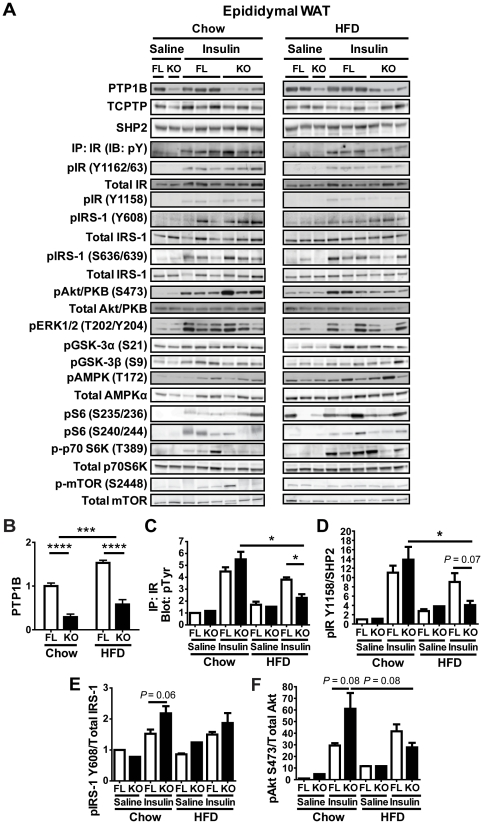
Reduced in vivo insulin signaling in epididymal white adipose tissue from HFD-fed adip-crePTP1B^−/−^ mice. *A*: Epididymal white adipose tissue immunoblots of insulin signaling components in chow- and HFD-fed fl/fl and adip-crePTP1B^−/−^ (KO) and fl/fl (FL) mice after injection with saline or insulin (10 mU/kg). *B*: PTP1B levels and deletion efficiency of fl/fl (*n* = 4–5) and adip-crePTP1B^−/−^ mice (*n* = 4) in epididymal white adipose tissue under chow- and HFD-fed conditions. Graphs C to F show phosphorylation levels of the indicated proteins in epididymal white adipose tissue after saline or insulin (10 mU/kg) injection of chow- or HFD-fed fl/fl and adip-crePTP1B^−/−^ mice, as indicated. Phosphorylated proteins were normalized as shown in the graphs. *C*: IP: IR (IB: pY). *D*: IR Y1158. *E*: IRS-1 Y608. *F*: Akt/PKB S473. White bar = fl/fl; black bar = adip-crePTP1B^−/−^. Data are represented as mean ± SEM; data were analyzed using two-way ANOVA with Bonferroni multiple comparisons post-tests to compare between diets, and two-tailed Student's t test to compare between different genotypes on the same diet (*P<0.05; **P<0.01; ***P<0.001; ****P<0.0001).

**Figure 5:**
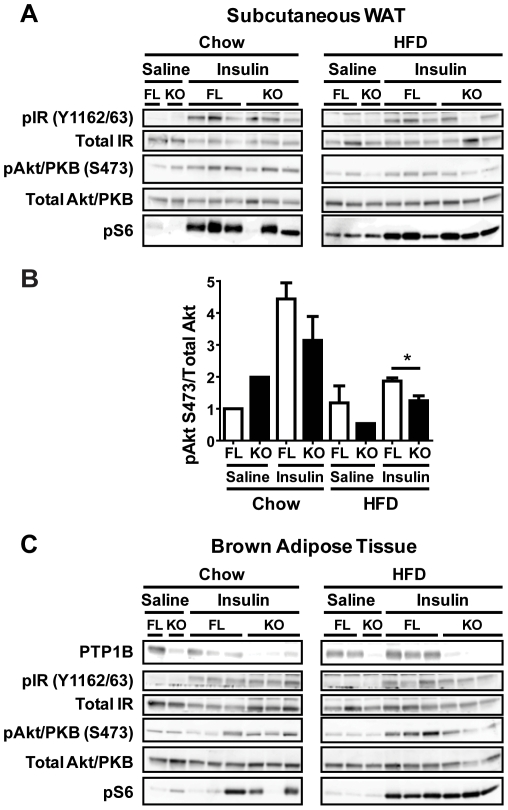
Reduced in vivo insulin signaling in subcutaneous white adipose tissue from HFD-fed adip-crePTP1B^−/−^ mice. *A*: Subcutaneous white adipose tissue immunoblots of insulin signaling components in chow- and HFD-fed fl/fl and adip-crePTP1B^−/−^ (KO) and fl/fl (FL) mice after injection with saline or insulin (10 mU/kg). *B*: Akt/PKB S473 phosphorylation levels normalized to total Akt/PKB in subcutaneous white adipose tissue after saline or insulin (10 mU/kg) injection of chow- or HFD-fed fl/fl (FL) and adip-crePTP1B^−/−^ mice (KO). *C*: Brown adipose tissue immunoblots of insulin signaling components in chow- and HFD-fed fl/fl and adip-crePTP1B^−/−^ (KO) and fl/fl (FL) mice after injection with saline or insulin (10 mU/kg). White bar = fl/fl; black bar = adip-crePTP1B^−/−^. Data are represented as mean ± SEM; data were analyzed using two-tailed Student's t test (*P<0.05; **P<0.01).

To investigate the molecular consequences of adipocyte-specific PTP1B deletion in WAT and BAT, mice were injected with either saline or insulin (10 mU/g body weight), and various components of the insulin signaling pathway were analyzed in epididymal-WAT (E-WAT), subcutaneous-WAT (SQ-WAT) and BAT. HFD-feeding of fl/fl controls led to significantly higher PTP1B levels in E-WAT (Figures 4A and B) and BAT (Figure 5C) compared with chow-fed fl/fl controls. In chow-fed mice, insulin-stimulated phosphorylation of the IR at sites Y1162/63 and Y1158, as well as total IR tyrosyl phosphorylation (measured following immunoprecipitation of the IR and anti-pY blotting) were comparable in E-WAT from both groups of mice (Figures 4A, C, D). However, phosphorylation of IRS-1 at site Y608 and Akt/PKB at site S473 trended to be higher in E-WAT from chow-fed adip-crePTP1B^−/−^ mice compared with controls (*P* = 0.06 and *P* = 0.08, respectively) (Figures 4A, E and F). Notably, relative to HFD-fed controls, insulin-evoked IR tyrosyl phosphorylation in E-WAT was significantly diminished in adip-crePTP1B^−/−^ mice on HFD (Figures 4A and C). There was a similar trend towards decreased phosphorylation at IR site Y1158 and pAkt/PKB site S473 in adip-crePTP1B^−/−^ mice (*P* = 0.07 and *P* = 0.08, respectively) (Figures 4A, D and F). There were no differences in the phosphorylation of the IR on site Y1162/63, IRS-1 (S636/639) ERK1/2 (T202/Y204), GSK-3α (S21), GSK-3β (S9), AMPK (T172), S6 ribosomal protein (S235/236), S6 ribosomal protein (S240/244), p70S6K (T389) or mTOR (S2448) on chow or HFD between adip-crePTP1B^−/−^ and control mice (Figure 4A). Total protein levels of other tyrosine phosphatases, such as TC-PTP and SHP2, were comparable in E-WAT of adip-crePTP1B^−/−^ and control mice on both diets (Figure 4A). In SQ-WAT the phosphorylation of Akt/PKB (S473) was similar on chow, but significantly decreased in HFD-fed adip-crePTP1B^−/−^ mice compared with controls (Figures 5A and B). However, there were no differences in SQ-WAT phosphorylation of the IR (Y1162/63) or S6 ribosomal protein (S235/236) between groups on both diets (Figure 5A). Furthermore, BAT phosphorylation levels of the IR (Y1162/63), Akt/PKB (S473) and S6 ribosomal protein (S235/236) were comparable between adip-crePTP1B^−/−^ and control mice on both diets (Figure 5C).

### No effect of adipocyte-PTP1B deletion on insulin signaling in isolated adipocytes

**Figure 6:**
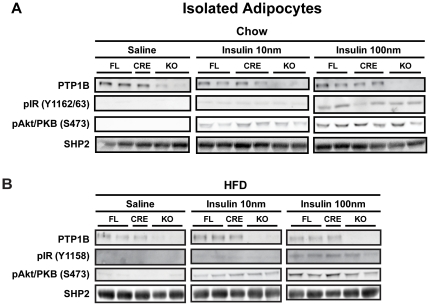
No effect of adipocyte-PTP1B deletion on insulin signaling in isolated adipocytes. *A*: Immunoblots of insulin-stimulated (0 nM, 10 nM or 100 nM insulin) isolated epididymal adipocytes from chow-fed control and adip-crePTP1B^−/−^ mice. *B*: Immunoblots of insulin-stimulated (0 nM, 10 nM or 100 nM insulin) isolated epididymal adipocytes from HFD-fed control and adip-crePTP1B^−/−^ mice.

To investigate whether the decreased insulin signaling observed in epididymal white adipose tissue *in vivo* was directly due to PTP1B deletion in adipocytes, insulin signaling stimulations were performed *ex vivo* on isolated adipocytes from control and adip-crePTP1B^−/−^ mice. Isolated adipocytes were stimulated with 0 nM, 1 nM (data not shown) 10 nM or 100 nM of insulin, and key components of the insulin signaling pathway, which were affected *in vivo*, were analyzed. There were no differences in the phosphorylation of the insulin receptor, Akt/PKB or S6 ribosomal protein between genotypes on either chow or HFD (Figures 6A and B).

### Increased lipogenic markers in HFD-fed adip-crePTP1B^−/−^ mice

**Figure 7:**
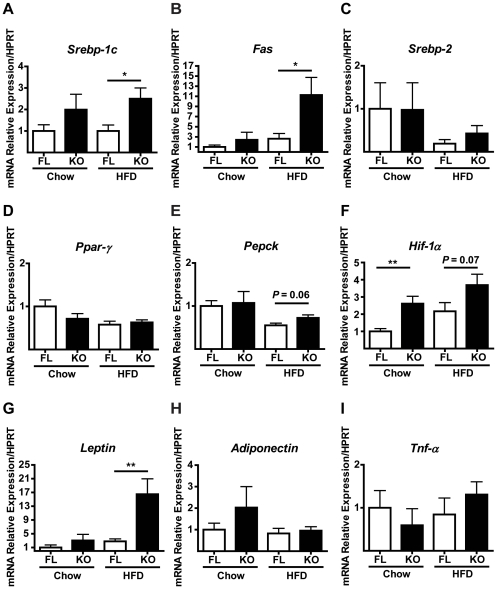
Increased lipogenic gene expression in HFD-fed adip-crePTP1B^−/−^ mice. Graphs A to I show relative mRNA levels of the indicated genes in epididymal WAT, measured by quantitative real-time PCR and normalized against *Hprt* mRNA. Chow-fed adip-crePTP1B^−/−^ (*n* = 4) and chow-fed fl/fl control mice (*n* = 13) were compared to HFD-fed adip-crePTP1B^−/−^ (*n* = 4) and HFD-fed fl/fl control mice (*n* = 15). *A*: *Srebp-1c*. *B*: *Fas*. *C*: *Srebp-2*. *D*: *Ppar-γ*. *E*: *Pepck*. *F*: *Hif-1α*. *G*: *Leptin*. *H*: *Adiponectin*. *I*: *Tnf-α*. White bar = fl/fl; black bar = adip-crePTP1B^−/−^. Data are represented as mean ± SEM; data were analyzed using two-tailed Student's t test (*P<0.05; **P<0.01).

The sterol regulatory element-binding proteins (SREBPs) coordinately activate the expression of over 30 genes involved in the uptake of fatty acids, triglycerides and phospholipids [40]. Expression of *Srebp-1c* and its target gene fatty acid synthase (*Fas*) were significantly higher in HFD-fed adip-crePTP1B^−/−^ mice compared with fl/fl controls (Figures 7A and B), consistent with increased basal lipogenesis (Figure 3C) and an increase in adipocyte size (Figure 1G and H). *Srebp2* and peroxisome proliferator-activated receptor gamma (*Ppar-γ*) expression levels were comparable between groups on both chow and HFD (Figures 7C and D). Phosphoenolpyruvate carboxykinase (*Pepck*) expression levels were comparable between groups on a chow diet but there was a trend (*P* = 0.06) on a HFD towards higher *Pepck* mRNA levels in adip-crePTP1B^−/−^ mice compared to fl/fl controls (Figure 7E). Hypoxia-inducible factor-1 alpha (*Hif-1α*) expression levels were significantly higher in adip-crePTP1B^−/−^ mice compared to fl/fl controls on a chow diet. As expected, HFD-feeding increased *Hif-1α* mRNA levels in both groups of mice, however there was a trend (*P* = 0.07) for HFD-fed adip-crePTP1B^−/−^ mice to have higher *Hif-1α* mRNA levels compared to fl/fl controls (Figure 7F). The mRNA expression of the adipokine *leptin* was significantly higher in adipose-tissue from HFD-fed adip-crePTP1B^−/−^ mice compared with fl/fl controls (Figure 7G), consistent with increased circulating serum leptin levels in these mice (Table 1). *Adiponectin* and *Tnf-α* gene expression were comparable between adip-crePTP1B^−/−^ mice and controls on both chow and HFD (Figures 7H and I).

## Discussion

Adipose-PTP1B^−/−^ mice were generated previously using the aP2-promoter in an attempt to evaluate the effect(s) of PTP1B on body mass control in adipose tissue. Despite only ∼50% reduction of PTP1B levels in white adipocytes, aP2-crePTP1B^−/−^ mice displayed significantly increased body weight on a HFD compared with PTP1B^fl/fl^ littermate controls [18]. However, whether adipocyte-specific PTP1B was the true cause of increased weight gain in these mice is unclear as the aP2-promoter cassette is also active in other cell types, such as macrophages, osteoblasts and cardiomyocytes [21], [22]. Here we used adiponectin-cre mice, which express Cre selectively in adipocytes [28] (Figure 1A).

As with muscle- and liver-specific PTP1B deletion, adipocyte-specific PTP1B deletion did not affect body weight or adiposity in mice fed chow or HFD. This would suggest that PTP1B deletion in other cell types might be the cause of the body weight effects observed in aP2-crePTP1B^−/−^ mice. It is also possible that the disparate weight differences between aP2-crePTP1B^−/−^ and adip-crePTP1B^−/−^ mice are partly due to different mouse backgrounds, as the aP2-crePTP1B^−/−^ mice were on a mixed 129Sv/J X C57BL/6J background whereas the adip-crePTP1B^−/−^ mice were backcrossed to C57BL/6 background for several generations.

Chow and HFD-fed adip-crePTP1B^−/−^ mice were found to have larger epididymal adipocytes than fl/fl controls. However, smaller adipocytes were observed in mice with a global PTP1B deletion compared with controls [16], [25]. This suggests that adipocyte-PTP1B deletion did not contribute to the previously observed decrease in fat mass and adipocyte size observed in global PTP1B^−/−^ mice; the latter most likely was caused by neuronal-PTP1B deletion [18].

Intriguingly, PTP1B has been shown to be involved in adipocyte differentiation. One study has shown that inhibitors resulting in PTP1B deficiency in fat, decreased genes involved in adipocyte differentiation [41]. Furthermore, a more recent study demonstrated that inhibition of PTP1B in 3T3-L1 adipocytes inhibits adipogenesis [42]. However, this does not appear to be the case in BAT. Two studies have demonstrated that PTP1B-deficiency in BAT promotes adipocyte differentiation and adipogenesis and protects against apoptosis-inducing stimuli [43], [44]. Therefore, in the current study, it is possible that PTP1B deletion has led to an inhibition of adipocyte differentiation in WAT, resulting in cells which have become enlarged to compensate for storing excess energy from both chow and HFD.

However, the mechanism causing this increase in adipocyte size is not clear. Another possibility for the increased cell size could be, at least partly, due to increased basal lipogenesis and elevated expression of *Srebp-1c* and *Fas*. Given that adip-crePTP1B^−/−^ mice have decreased insulin signaling on HFD, and that leptin normally inhibits lipogenesis by stimulating fatty acid oxidation via negative regulation of *Srebp-1c* [37], [38], it is surprising that *Srebp-1c* and *Fas* gene expression are increased compared with controls. Interestingly however, an opposite and similarly paradoxical phenotype was observed in liver-specific PTP1B knockout mice: they displayed increased hepatic insulin signaling and decreased expression levels of hepatic *Srebp-1c*, *Fas* and other lipogenic markers [5], [45]. In the liver, PTP1B may regulate *Srebp-1a* and *Srebp-1c* mRNA expression via phosphatase 2A (PP2A) activity [46]. It is suspected that PTP1B may affect *Srebp-1* gene expression via a non-insulin signaling pathway in the liver [47], which might also be the case in adipocytes.

Interestingly, *in vivo* over-expression of *Pepck* in adipose tissue was shown to increase glyceroneogenesis and fatty acid re-esterification, leading to increased adipocyte size and fat mass [48]. Mice with an adipocyte-PTP1B deletion displayed increased expression of *Pepck* on a HFD compared with fl/fl controls, suggesting that the increased adipocyte size may also be partly due to increased glyceroneogenesis and fatty acid re-esterification in these mice.

During the early stages of obesity, hypoxic conditions cause an increase in the level of *Hif-1α* expression in mice on a HFD and in genetically obese *ob/ob* mice [49]. *Hif-1α* over-expression *in vivo* has been shown to lead to increased adipocyte cell size and it has been proposed that *Hif-1α* upregulation represents one of the earliest events in adipose tissue expansion and dysfunction [49]. Indeed, HFD-feeding led to increased *Hif-1α* expression in both groups of mice. Interestingly however, we found that *Hif-1α* expression was higher in chow and HFD-fed adip-crePTP1B^−/−^ mice compared with fl/fl controls, suggesting that these mice are more prone to hypoxia-induced adipose tissue expansion, which may have also contributed to enlarged adipocytes and increased leptin secretion. Furthermore, a recent study which demonstrated that HIF binds to the PTP1B promoter and reduces PTP1B expression, and proposed that there was a HIF-regulated VHL-PTP1B-Src signaling axis in renal cell carcinoma (RCC) cells, suggests that there is a relationship between *HIF-1* expression and PTP1B signaling [50].

The expression and release of leptin have been shown to depend on adipocyte cell size in rodents and humans [51], [52]. In HFD-fed mice, adipocyte-specific PTP1B deletion increased serum leptin levels seven-fold and *leptin* mRNA expression six-fold in comparison to fl/fl controls, which may be due to the increased volume of adipocytes in these mice, as body weight and *ad libitum* food intake were comparable between HFD-fed adip-crePTP1B^−/−^ and control mice. An increase in leptin secretion was also observed in neuronal-specific PTP1B knockout mice, which displayed increased serum leptin levels but remained leptin hypersensitive [18]. Reduced suppression of food intake in response to exogenous leptin in HFD-fed adip-crePTP1B^−/−^ mice suggests that they were more leptin resistant than their HFD-fed controls. PTP1B clearly appears to play an important role in leptin secretion but the pathways involved remain to be determined.

Adipose tissue has been shown to play an important role in glucose homeostasis via secretion of adipokines and accounts for ∼10–15% of postprandial glucose uptake [53]. Large adipocytes exhibit reduced glucose uptake and are less insulin-sensitive than small adipocytes as they become enriched with large lipid droplets [54]. Fasted blood glucose levels after 8 and 14 weeks of HFD, and HOMA-IR after 14 weeks HFD, were significantly higher in adip-crePTP1B^−/−^ mice than fl/fl controls suggesting mild glucose intolerance in these mice. However, adip-crePTP1B^−/−^ mice showed no alteration in serum adipokines (other than leptin), despite larger adipocytes and increased lipogenesis.

Reports of the effect(s) of adipose tissue PTP1B-deficiency on insulin signaling are conflicting. *In vivo* antisense oligonucleotide treatment, which decreased adipose-PTP1B, elicited some improvements in insulin signaling [26], [27]. However, over-expression of PTP1B in differentiated adipocytes had minimal effects on insulin signaling [23]. Furthermore, adipose tissue-insulin signaling was not different in global PTP1B^−/−^ mice relative to controls [17]. In contrast, in another study, basal hyper-phosphorylation of p70S6K was observed in the adipose tissue of globally PTP1B-deficient mice, which was described as the cause of decreased insulin-stimulated phosphorylation of IRS-1 and decreased activity of Akt/PKB, leading to adipose-specific insulin resistance in PTP1B^−/−^ mice [25]. Consistent with the latter studies, we show here that insulin-stimulated phosphorylation of IR and Akt/PKB, under HFD-feeding conditions, is impaired in mice with an adipocyte-specific PTP1B deletion. Interestingly, leptin has been shown to impair insulin signaling in rat adipocytes [55], which is also consistent with these observations. It is possible that the increases in serum leptin, in the absence of PTP1B in adipocytes, and the potential of signaling crosstalk, may be confounding detection of predicted changes in insulin signaling *in vivo*. However, since we did not observe any differences between groups in IR phosphorylation, or that of the downstream components, in isolated adipocytes under different insulin concentrations, this confirms our *in vivo* findings that PTP1B does not appear to be the main IR phosphatase nor be a negative regulator of insulin signaling in adipocytes. In addition, adipocyte-PTP1B deletion did not result in any differences in the phosphorylation of p70S6K, S6 ribosomal protein, IRS-1, mTOR or a number of other insulin signaling pathway components in our studies. Such differences in findings may be due to altered cross-talk between the adipose tissue and other tissues, such as the liver and the central nervous system, in global PTP1B^−/−^ mice.

We therefore suggest that PTP1B deletion in adipocytes enhances basal lipogenesis and fatty acid re-esterification via a non-classical insulin signaling pathway, by increasing lipogenic (*Srebp-1c*, *Fas*) and gluconeogenic (*Pepck*) mRNA expression, which subsequently leads to increased lipid storage and enlarged adipocytes. This increased adipocyte size, combined with increased *Hif-1α* gene expression, results in hypoxia-induced adipose tissue dysfunction and leads to augmented leptin secretion, which dampens adipocyte-insulin signaling; finally leading to leptin resistance and mildly elevated fasting blood glucose. Our findings and those of Ruffolo *et al.* suggest that some compensatory mechanism(s), such as the up-regulation of another protein tyrosine phosphatase may be involved in regulating adipocyte-insulin signaling [17], [25]. T-cell protein-tyrosine phosphatase (TC-PTP) is a ubiquitous tyrosine-specific phosphatase with a high degree of similarity to PTP1B [56]. Furthermore, TC-PTP has been shown to coordinately regulate insulin signaling with PTP1B and act to control common and distinct insulin signaling pathways within the same cells [56]. In the current study, TC-PTP was not up-regulated by PTP1B deletion. Similarly, no changes in SHP2 levels were observed in adip-crePTP1B^−/−^ mice. However, it is still possible that some other protein tyrosine phosphatase(s) may play a role as a negative regulator of IR signaling in adipocytes.

Overall, tissue-specific knockout studies of PTP1B have revealed key roles for brain-, liver- and muscle-PTP1B in the regulation of global energy and glucose homeostasis. However, adipocyte-PTP1B primarily appears to only locally regulate lipogenic gene expression, adipocyte cell size, and leptin secretion and does not appear to play a major role in regulating body weight/adiposity or whole body glucose homeostasis.

## Supporting Information

Figure S1 **PTP1B deletion of fl/fl control, adip-cre-alone control and adip-crePTP1B^+/−^ mice.** PTP1B deletion efficiency of HFD-fed fl/fl (FL), adip-cre-alone (CRE) and adip-crePTP1B^+/−^ (HET) mice in epididymal WAT.
(TIF)

Figure S2 **Saline control injections to leptin sensitivity experiment.** No significant effect on food intake after saline control injections in chow fed adip-crePTP1B^−/−^ and fl/fl control mice (*n* = 3 mice/group). White circles = chow fl/fl saline; black circles = chow adip-crePTP1B^−/−^ saline. Data are represented as mean ± SEM.
(TIF)

Figure S3 **Area under the curve of** **Figure 2B** **.** No significant differences between groups. PTP1B deletion does not significantly affect glucose clearance following a glucose bolus. White bars = fl/fl; diagonally striped bars = adip-cre; horizontally striped bars = adip-crePTP1B^+/−^; black bars = adip-crePTP1B^−/−^. Data are represented as mean ± SEM.
(TIF)

Figure S4 **Adipocyte morphology in subcutaneous and peri-renal fat pads.** No differences of subcutaneous or peri-renal adipocyte cell size between HFD-fed adip-crePTP1B^−/−^ mice (KO) and fl/fl (FL) control mice. *n* = 4 mice/group. White bars = fl/fl; black bars = adip-crePTP1B^−/−^. Data are represented as mean ± SEM.
(TIF)

Table S1 **Real time quantitative PCR primer sequences for gene expression analysis and PCR primer sequences for genotyping.**
(DOCX)
