# Correction: Adipocyte-Specific Protein Tyrosine Phosphatase 1B Deletion Increases Lipogenesis, Adipocyte Cell Size and Is a Minor Regulator of Glucose Homeostasis

**DOI:** 10.1371/annotation/c543ef23-f1b1-4bc7-9593-32387653bc05

**Published:** 2012-05-29

**Authors:** Carl Owen, Alicja Czopek, Abdelali Agouni, Louise Grant, Robert Judson, Emma K. Lees, George D. Mcilroy, Olga Göransson, Andy Welch, Kendra K. Bence, Barbara B. Kahn, Benjamin G. Neel, Nimesh Mody, Mirela Delibegović

Some information was missing from the Funding section. The missing information is: BBK and BGN are funded by NIH R01 DK060838.

