# Supplementary figures and images for: Adipocyte-Specific Protein Tyrosine Phosphatase 1B Deletion Increases Lipogenesis, Adipocyte Cell Size and Is a Minor Regulator of Glucose Homeostasis
Source: PLoS One. 2012 Feb 28;7(2):e32700. doi: 10.1371/journal.pone.0032700 (PMC3289674; doi:10.1371/journal.pone.0032700)

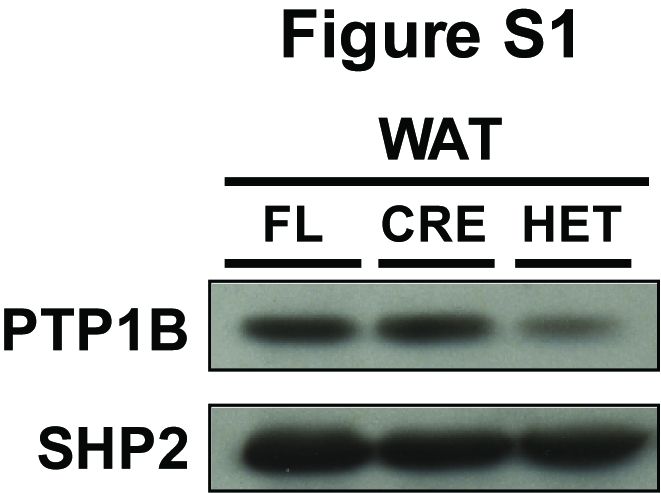

Supplement: Figure S1 — PTP1B deletion of fl/fl control, adip-cre-alone control and adip-crePTP1B+/− mice. PTP1B deletion efficiency of HFD-fed fl/fl (FL), adip-cre-alone (CRE) and adip-crePTP1B+/− (HET) mice in epididymal WAT. (TIF) [file pone.0032700.s001.tif]

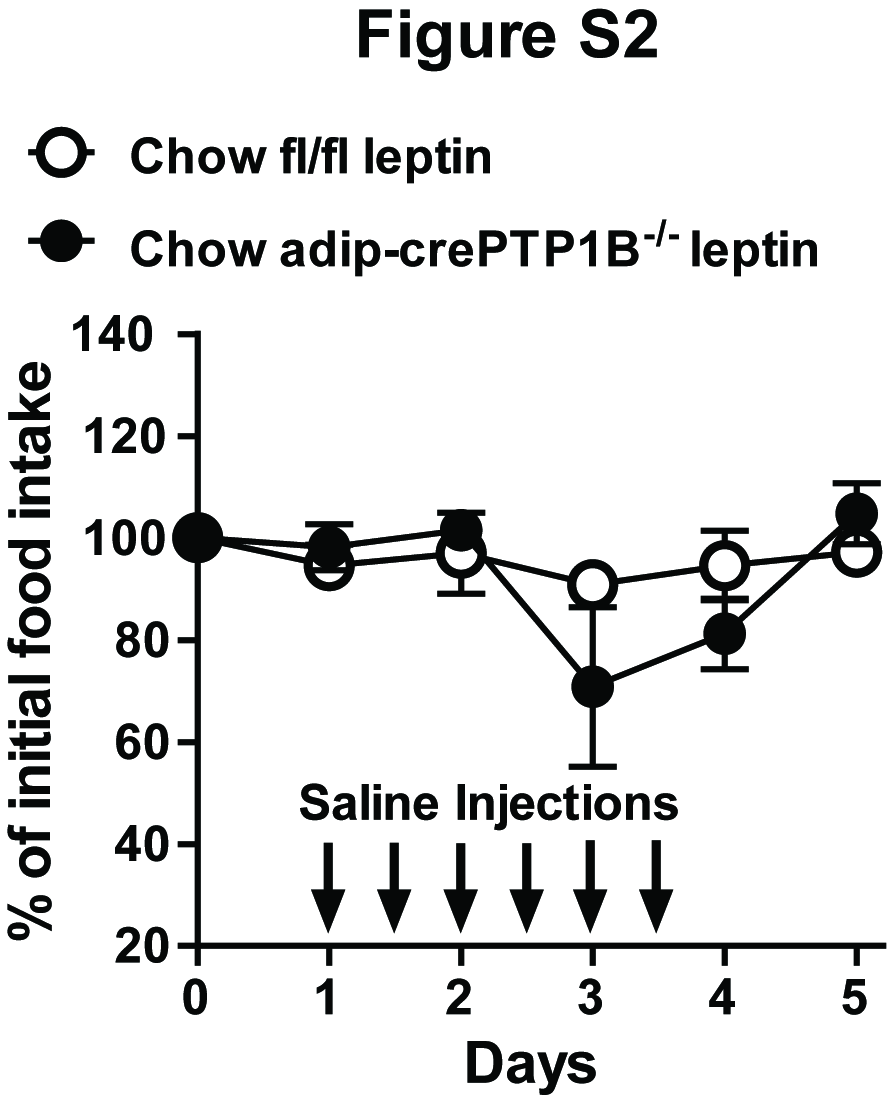

Supplement: Figure S2 — Saline control injections to leptin sensitivity experiment. No significant effect on food intake after saline control injections in chow fed adip-crePTP1B−/− and fl/fl control mice (n = 3 mice/group). White circles = chow fl/fl saline; black circles = chow adip-crePTP1B−/− saline. Data are represented as mean ± SEM. (TIF) [file pone.0032700.s002.tif]

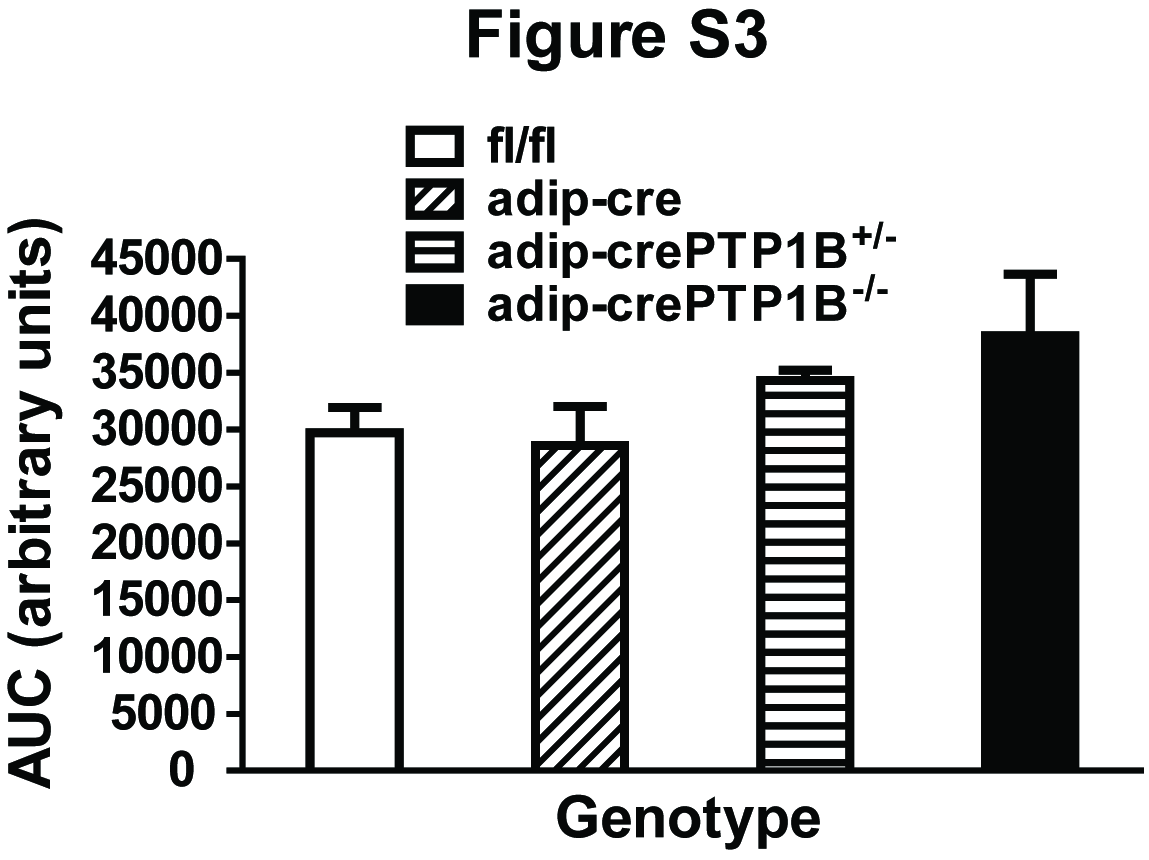

Supplement: Figure S3 — Area under the curve of Figure 2B . No significant differences between groups. PTP1B deletion does not significantly affect glucose clearance following a glucose bolus. White bars = fl/fl; diagonally striped bars = adip-cre; horizontally striped bars = adip-crePTP1B+/−; black bars = adip-crePTP1B−/−. Data are represented as mean ± SEM. (TIF) [file pone.0032700.s003.tif]

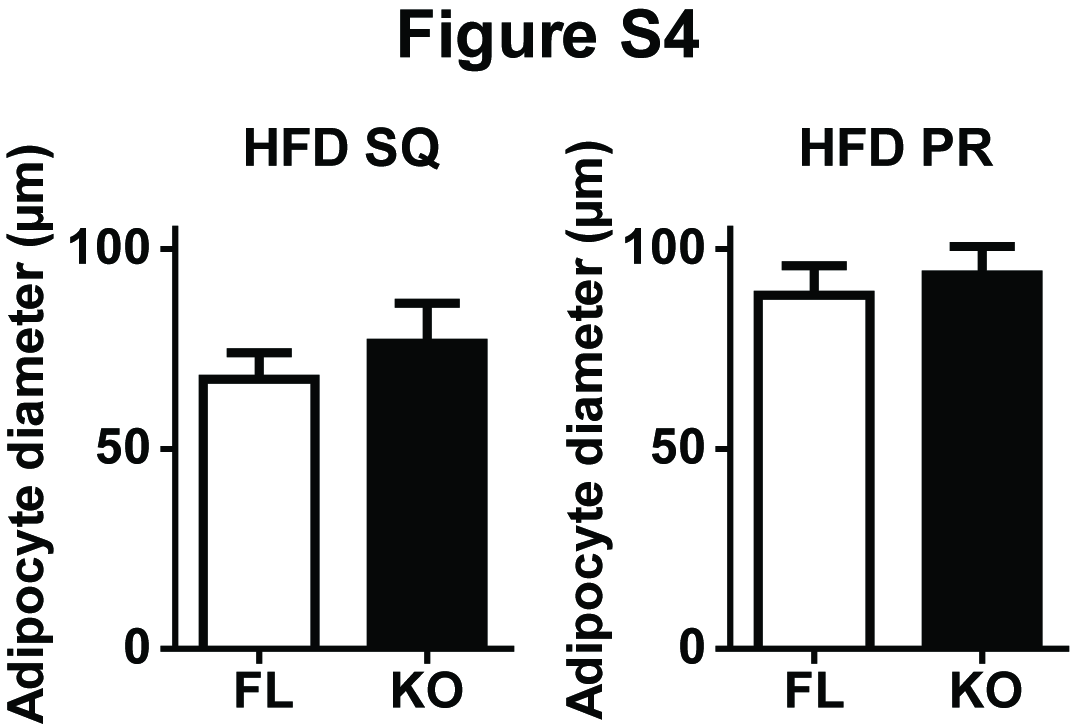

Supplement: Figure S4 — Adipocyte morphology in subcutaneous and peri-renal fat pads. No differences of subcutaneous or peri-renal adipocyte cell size between HFD-fed adip-crePTP1B−/− mice (KO) and fl/fl (FL) control mice. n = 4 mice/group. White bars = fl/fl; black bars = adip-crePTP1B−/−. Data are represented as mean ± SEM. (TIF) [file pone.0032700.s004.tif]
