# Supplementary material for: Adipocyte-Specific Protein Tyrosine Phosphatase 1B Deletion Increases Lipogenesis, Adipocyte Cell Size and Is a Minor Regulator of Glucose Homeostasis
Source: PLoS One. 2012 Feb 28;7(2):e32700. doi: 10.1371/journal.pone.0032700 (PMC3289674; doi:10.1371/journal.pone.0032700)
Supplement: Table S1 — Real time quantitative PCR primer sequences for gene expression analysis and PCR primer sequences for genotyping. (DOCX) [file pone.0032700.s005.docx]

**SUPPLEMENTAL DATA**

TABLE S1. Real time quantitative PCR primer sequences for gene expression analysis and PCR primer sequences for genotyping.

| **Gene** | **Forward** | **Reverse** |
| --- | --- | --- |
| **qPCR Primers** |  |  |
| *Srebp-1c* | ACGGAGCCATGGATTGCACATTTG | AGGCTGTAGGATGGTGAGTGG |
| *Fas* | AAGCTCAGTGTGCCCACCTA | ATGGCAACGTGACACTGCTG |
| *Srebp-2* | GCAGCAACGGGACCATTCT | CCCCATGACTAAGTCCTTCAACT |
| *Ppar-γ* | TGAAAGAAGCGGTGAACCACTG | TGGCATCTCTGTGTCAACCATG |
| *Pepck* | GAGATAGCGGCACAAT | TTCAGAGACTATGCGGTG |
| *Hif-1α* | ACCTTCATCGGAAACTCCAAAG | CTGTTAGGCTGGGAAAAGTTAGG |
| *Leptin* | GAGACCCCTGTGTCGGTTC | CTGCGTGTGTGAAATGTCATTG |
| *Adiponectin* | TGTTCCTCTTAATCCTGCCCA | CCAACCTGCACAAGTTCCCTT |
| *Tnf-α* | CCCTCACACTCAGATCATCTTCT | GCTACGACGTGGGCTACAG |
| **PCR Primers** |  |  |
| *General Cre* | AAATGGTTTCCCGCAGAACC | TAGCTGGCTGGTGGCAGATG |
| *Ptp1b Floxed* | TGCTCACTCACCCTGCTACAA | GAAATGGCTCACTCCTACTGG |
|  | | |
